# Molecular Mechanisms Contributing Bacterial Infections to the Incidence of Various Types of Cancer

**DOI:** 10.1155/2020/4070419

**Published:** 2020-07-08

**Authors:** Salah A. Sheweita, Awad S. Alsamghan

**Affiliations:** ^1^Department of Biotechnology, Institute of Graduate Studies and Research, Alexandria University, Alexandria 21526, Egypt; ^2^Department of Clinical Biochemistry, Faculty of Medicine, King Khalid University, Abha, Saudi Arabia; ^3^Department of Family and Community Medicine, Faculty of Medicine, King Khalid University, Abha, Saudi Arabia

## Abstract

Cancer causes a major health concern worldwide due to high incidence and mortality rates. To accomplish this purpose, the Scopus, PubMed, and Web of Science databases were searched using the keywords bacteria and cancer. Most of published research addressed several different factors that induced cancer, such as toxins, medications, smoking, and obesity. Nonetheless, few studies are dealing with cancer induction via bacterial infection. In addition, mechanisms of cancer induction via bacterial infections are not well understood. Therefore, in this review, we will shed light on different bacteria that induced cancer via different molecular mechanisms. Among the bacterial infection that induced cancer, *Helicobacter pylori* was the first recognized bacteria which caused gastric cancer and might be also linked to extragastric cancer in humans. *H. pylori* has been associated with adenocarcinoma in the distal stomach by its ability to cause severe inflammations. It has been found that inflammations induced cancer via different mechanisms including induction of cell proliferation and production of high levels of free radicals. Recently, free radicals were found to induce and cause various types of cancer. *Salmonella typhi* has been found to be associated with gallbladder carcinoma (GBC). Also, intercellular infection of lungs with Chlamydia pneumoniae was found to contribute as one of the ethological factors of lung cancer. Moreover, infection of the urinary tract with *Staphylococcus aureus*, *Klebsiella* spp., and *Proteus mirabilis* has been found to cause bladder cancer. These microorganisms produce a high level of N-nitrosamines which are metabolically activated leading to the generation of alkylating agents that damage DNA and other macromolecules. It is concluded that a certain bacterium is linked with induction of a specific type of cancer via different molecular and biochemical mechanisms as discussed in the text in details. This infection could potentially affect human health in different ways. In addition, it is important to know the possible factors involved in cancer induction for better treatment of cancer patients.

## 1. Introduction

**Figure 1:**
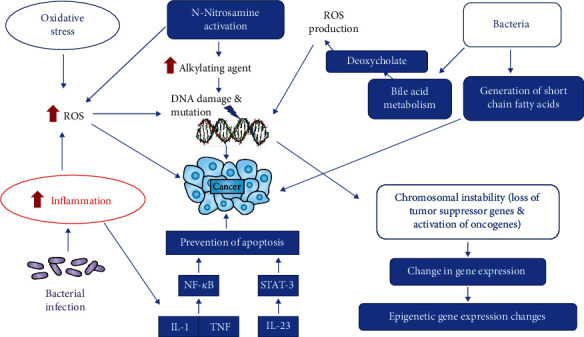
Different molecular mechanisms of carcinogenesis induced by bacterial infection and oxidative stress.

Genetic, environmental, and dietary factors are identified as the main factors of cancer induction, and their interaction leads to carcinogenesis. Environmental factors such as tobacco smoke and occupational exposure to hazardous chemicals account for 90% of all cancers. The majority of the exogenous compounds are chemical carcinogens which undergo metabolic activation to form metabolites which interact with cellular macromolecules and initiate carcinogenesis by causing damage to the DNA, hence are called exogenous genotoxic carcinogens. These carcinogens consist of a wide variety of compounds, which differ in their chemical structure but possess a common ability to form chemical bonds with DNA, resulting in the generation of “DNA adducts.” The formation of these DNA adducts is recognized as the initial step in chemical carcinogenesis [1, 2]. In addition, the initial stage of gene mutilation is also based on endogenous mechanisms that cause mutations or even gene deletions. Very common endogenous mediators are free radicals or reactive oxygen species (ROS), which cause oxidative damage to DNA and cause different mutations (Figure 1) [3, 4]. Recent studies have shown a causative relationship between bacterial infection and the onset of cancer in organs such as lungs, colon, and cervix, which are constantly exposed to bacteria (Figure 1) [5]. The harmony of cells and the control of its growth and proliferation are regulated by a well-synchronized signalling pathway. Any alteration or deregulation of these pathways triggers carcinogenesis. During bacterial infection, various bacteria target and trigger these signalling pathways. Therefore, in this review, we have focused on the role of some bacteria in the incidence of cancer since a number of bacteria have been associated with cancer via triggering signalling pathways. *Helicobacter pylori*, *Salmonella typhi*, *Streptococcus bevies*, and *Chlamydia pneumonia* cause stomach, gallbladder, colorectal, and lung cancers, respectively, through different mechanisms.

### 1.1. *Salmonella typhi* Induced Gallbladder Cancer

*Salmonella typhi* is a rod-shaped gram-negative bacterium of the Enterobacteriaceae family, which is well known to cause typhoid or enteric fever. These bacteria colonize in the gallbladder causing asymptomatic chronic infection [6]. Epidemiological studies from *S. typhi* endemic regions have shown that most of the chronically infected carriers developed gall cholelithiasis, a primary predisposing reason for the onset of gallbladder cancer (GBC). Typhoid toxins produced by *S. typhi* have a carcinogenic potential which damages the DNA and alters the cell cycle in the infected cells. Apart from this, the extracellular polymeric substances (EPS) produced in the biofilm of *S. typhi* are the key factor for persistent infection and cholecystitis leading to exposure of the epithelium to carcinogenic toxins produced by *S. typhi* [6]. Until the 1990s, in Chile, under the backdrop of a typhoid epidemic, a high prevalence of gallbladder cancer incidence was observed, which was mainly attributed to its association with Salmonella enterica serovar typhi (S. typhi) Vi antibodies. However, the exact mechanism underlying this association is still under investigation [7]. Another study in India claimed chronic Salmonella enterica serovar typhi infection to be a significant risk factor for the development of gallbladder cancer, although no direct association and mechanism have been explained yet [8].

#### 1.1.1. Molecular Mechanism Underlying Induction of Gallbladder Cancer

The role of *Salmonella typhi* in altering the genomic sequence of tumor protein p53 (TP53) and amplification of protooncogene c-MYC results in malignant transformation from predisposed mice gallbladder organoids and fibroblasts (Table 1 and Figure 1). *Salmonella typhi* effectors released during an infection contribute to the autoactivation of protein kinase which is triggered by mitogen (MAPK) and the Akt pathways (Table 1). This process is pathognomonic in initiating as well as sustaining malignant transformation, which is a consistent observation in gallbladder cancer patients in India. Hence, the role of *Salmonella typhi* predisposed epithelium of gallbladder to toxic metabolites was established [8]. Another mechanism is primarily attributable to the production by *S. typhi* of genotoxic substances (cytolethal distending toxin B (CdtB)), which is the functional unit of cytolethal distending toxin (CDT) and cytotoxic necrotizing factor 1 (CNF1). The CdtB works by targeting the DNA in the human host cells while CNF1 inhibits the activity of cytokines leading to inflammation and cell cycle inhibition [9] (Table 1 and Figure 1). Moreover, CNF1 also affects the transcription termination process in prokaryotes by altering the Rho proteins [9]. *S. typhi* changed the protein expressions of CdtB and CNF1 leading ultimately to cancer of the gallbladder.

The function of the gallbladder is to store bile, which consists of bile salts and acids. Various mechanisms have been suggested in which altered bile salt metabolism produces carcinogenic compounds from long-term S. typhi carriers. Bacterial enzymes which were present in *S. typhi* work primarily on bile acids to generate high concentrations of toxic metabolites and secondary bile acids (Figure 1). These toxic metabolites lead to the pathology of the epithelium of the gallbladder. A glycosidase enzyme, *β*-glucuronidase, deconjugates the conjugated primary bile salts resulting in the production of high concentrations of toxic metabolites which have carcinogenic effects [9] (Table 1). These metabolites bind to DNA in human epithelial cells, exerting its mitogenic action [10, 11]. It has also been shown that high concentrations of biliary deoxycholate, a secondary bile acid, are present at elevated levels in patients with gallbladder carcinoma [9]. Another mechanism suggested that *S. typhi* mutagenicity is due to its cholesterol interaction, which ultimately forms the structural basis of the gallstones. These bacteria not only transform the bile salts into secondary forms of bile but also convert cholesterol itself into carcinogenic compounds like 5-alpha,6-alpha-epoxide cholesterol, leading to pathogenic changes in epithelial cells [12] (Table 1). Another study has shown that *S. typhi* bacteria are capable of metabolizing primary bile acids into mutagenic cholic acid derivative types in the presence of bile and cholesterol substrates that cause gall balder cancer [11].

### 1.2. *Chlamydia pneumonia* and Lung Cancer

Lung cancer is closely related to chronic inflammation, but it has not completely elucidated the causes of inflammation and the basic immune mediators. Chlamydophila (formerly called Chlamydia) pneumoniae is a species of Chlamydia, an intracellular bacterium that infects the cells of the respiratory tract in humans. It is responsible for about 10% of community-acquired pneumonia and 5% of sinusitis, pharyngitis, and bronchitis [13]. Chronic pneumocyte infection by C. pneumoniae predisposes for the development of lung cancer, which is a major public health concern due to its high incidence and mortality [13]. The existence of C. pneumoniae in 230 lung cancer cases has been found, and the lung cancer risk was increased by 1.6 in C. pneumoniae-infected patients [14]. In addition, in patients with bronchoscopy and lung cancer, an association between chronic C. pneumoniae infection and incidence of lung cancer was found [15]. It has been found that IgA antibodies were increased in lung cancer patients infected with C. pneumoniae [16]. In another study, increased C. pneumoniae-specific IgA levels in smokers with lung cancer were found [17].

#### 1.2.1. Mechanism of Lung Cancer Induction

Post chlamydial infection, numerous proteins are released which are hypothesized to cause lung cancer by targeting either mitochondrial or cytoplasmic cellular activities. Their mechanism of action is by competitive inhibition where these targeting proteins and host proteins compete to bind with the substrate [5]. In a study done by Alshamsan et al., it was reported that out of 1112 proteins derived from C. pneumoniae, 183 and 513 proteins targeted the mitochondrial and cytoplasmic cellular processes of the host cells, respectively [18]. This leads to disturbance of normal cellular growth, resulting in an alteration in apoptosis or programmed cell death leading to the development of lung cancer. It is also assumed that these proteins are incorporated into the intracellular organelles leading to the formation of a host cell proteome. Various bioinformatics tools such as nuclear localization signal (NLS) mapper, Hum-mPLoc 2.0, ExPASy pI/Mw tool, and balanced subcellular localization predictor (BaCeILo) showed that 47 of the 1112 bacterial proteins were responsible for nuclear targeting which altered the host replication and transcription [5].

Many evidences have showed that microRNA (miRNA) played an important role in the metastasis and progression of lung cancer. A miRNA, miR-328, targets H2AX (a histone protein) in the regulation of lung cancer cell apoptosis (Table 1). Overexpression of miRNA-328 is associated with lung cancer, whereas its downregulation was shown to decrease the incidence of lung cancer that is induced by C. pneumoniae. In addition, suppression of miRNA-328 causes increased caspase 3 activity and apoptosis in cancer cells, resulting in lesser tumor volume [19].

A diverse bacterial community is colonized in the lung mucosal tissue and is commonly linked to clinical outcomes in patients with lung cancer which triggers lung adenocarcinoma-related inflammation by stimulating lung-resident *γδ* T cells [20]. It has been found that commensal bacteria stimulated the development of Myd88-dependent IL-1b and IL-23 from myeloid cells, inducing the proliferation and activation of Vg6+Vd1+*γδ* T cells containing IL-17 and other effector molecules to promote inflammation and proliferation of tumor cells (Figure 1) [20]. Local microbiota-immune crosstalk has been found to be correlated with the development of lung tumors, and molecular mediators that can serve as effective targets for lung cancer intervention have been identified (Table 1) [20]. Other mechanisms of bacterial infection causing lung cancer are the gene damage and the neoplastic transformation which are triggered by inflammatory mediators, nitric oxide (NO), and other reactive oxygen species (Figure 1) [21, 22]. An in vitro study has shown that chronic bacterial infection by *C. pneumoniae* causes increased secretion of cytokines, IL-8, IL-10, and TNF, in human alveolar macrophages and peripheral blood mononuclear cells (Table 1 and Figure 1) [23]. IL-8 is an angiogenic factor acting as a promotor of tumor growth of non-small-cell lung carcinomas in humans [24, 25]. Moreover, nitric oxide liberation is also increased in chlamydial infections which might induce an inflammatory response that leads to the development of lung cancer [26].

### 1.3. Helicobacter pylori and Gastric Cancer

*Helicobacter pylori* (*H. pylori*) is a gram-negative microaerophilic bacterium having a helical shape with an ability to penetrate the mucoid lining of the stomach causing infection. Although many patients infected with *H. pylori* may be asymptomatic, long-term complications include gastric ulcers, inflammation of the gastric lining leading to gastritis, and gastric carcinoma. Gastric carcinoma is a global health concern due to high morbidity and mortality. Though there are numerous predisposing risk factors such as family history, dietary habits (high salts and nitrates), alcohol, and smoking, *H. pylori* infection has one strongest association with gastric carcinoma [27]. Apart from environmental, dietary, and genetic factors which play a vital role in the development of most of the cancers, infection by *H. pylori* also causes cancer [28, 29]. The ability of the bacteria to enter the gastric cells and colonize for years allows it to interact with human cells and impart its harmful effects. These microbes release an oncoprotein, CagA, which affects the normal epithelial cell division in the gastric mucosa (Table 1) [30]. Other factors such as environmental, dietary (essential micronutrients), and gastrointestinal microflora also increase the carcinogenic potential of *H. pylori* [30]. In order to study the association between *H. pylori* and gastric carcinoma, epidemiological studies were carried out since 1991. Numerous studies on a large number of individuals were conducted, and reports were published [31–33]. These studies provided valuable insights and strongly encouraged subsequent studies. Later, in another study, it was demonstrated that *H. pylori* antibodies were highly prevalent in asymptomatic patients who developed gastric carcinoma [34, 35]. Nolen et al. reported an earlier onset of gastric carcinoma and a higher mortality rate in Alaska Native people as compared to the US population. This was attributed to the higher prevalence of *H. pylori* infection, antimicrobial resistance, and reinfection in people in Alaska Natives as compared to the US population [36]. A study in Japan on 213 gastric cancer patients showed 88.2% of patients having higher levels of *H. pylori* antibodies. This too suggests the role of *H. pylori* in various preceding stages such as acute gastritis, atrophic gastritis, intestinal cell metaplasia, and finally intestinal type of gastric carcinoma [37]. Another study by Frydman et al. showed a strong relationship between *H. pylori* infection and gastric cancer in younger gastric carcinoma patients [38].

#### 1.3.1. Mechanisms of Induction of Gastric Carcinoma

Patients with a history of *H. pylori* infection underwent annual endoscopic observation after eradication and were screened for novel markers such as gastric epithelium released tissue, protein biomarkers, and other proteins associated with cellular metaplasia in dysplasia in carcinoma. Other markers such as CD44, a signalling molecule in cell proliferation and differentiation, and metallopeptidase, an immune response-mediated marker, were also assayed (Table 1) [39, 40]. Metaplastic intestinal cells are characterized by overexpression of gastrointestinal stem cell markers like PROM1 gene product, leucine-rich repeat-containing G-protein coupled receptor 5 (LGR5), and genes related to the metabolism of messenger RNA and nucleic acids [39].

Various mechanisms have explained the role of *H. pylori* in altering the chemical contents of gastric juice and changes in gastric mucosal cells which cause chronic inflammation and subsequent carcinogenesis [41]. Colonization of gastric mucosal cells by *H. pylori* results in an inflammatory response by host cells. This leads to infiltration of host cells by macrophages, polymorph, nuclear leukocytes, T lymphocytes, and B lymphocytes. Therefore, the pathogen, along with its induced cytokines, stimulates the accumulation and activation of inflammatory cells [42]. Infiltration of gastric mucosal cells by activated inflammatory cells and neutrophils following infection by *H. pylori* produces free oxygen radicals both in the pyloric and duodenal mucosa which upregulates the production of IL-8 resulting in a greater inflammatory response [43–46]. These free radicals, being highly reactive, cause damage to proteins and DNA resulting in mutations (Table 1) [47]. Data showing the role of free radicals in the onset of gastric carcinoma is lacking [48]. Post *H. pylori* infection results in increased ammonia levels in the gastric mucosal cells of rats, which acted as promotors of gastric carcinoma by inducing N-methyl-N-nitro-N-nitroso guanidine (MNNG) [49]. Ascorbic acid, an antioxidant, reacts with nitroso compounds producing nitric oxide instead of harmful N-nitroso compounds [50]. Ascorbic acid concentration is lowered in the gastric juice following H. pylori infection, resulting in increased activity of free intermediate radicals. However, following eradication of *H. pylori*, increased gastric juice levels of vitamin C were observed [51–53]. As with most of the other microbes, the oncogenic activity in *H. pylori* is attributed to its proteins. Expression of cytotoxic mediators such as CagA and VacA by *H. pylori* causes activation or differentiation of gastric fibroblasts in rats which disrupt multiple cell signalling and proliferation pathways. Some major pathways include deregulation of Janus kinase/signal transducers, activation of nuclear factor kappa B (NF-*κ*B), and activation of transcription (JAK/STAT), which lead to inflammation and initiation of carcinogenic cascades (Table 1) [54]. Although the inflammatory process begins in the epithelial cells, it spreads to the surrounding activated fibroblast cells resulting in tumor progression, invasion, and metastasis. The expression of downstream targets of STAT3 and the epithelial-mesenchymal transition inducing transcription factor (EMT-TFs) are increased in activated fibroblasts (Figure 1) [54, 55].

AMP-activated alpha 1 catalytic subunit (PRKAA1) is one of the subunits of the mammalian 5′-AMP-activated protein kinase (AMPK). They play a crucial role in the maintenance of intracellular energy metabolism and hence are considered as a gastric carcinoma risk factor [56]. In NF-*κ*Bp50 knockdown rats, *H. pylori* infection upregulates the expression of p-NF-*κ*Bp50, NF-*κ*Bp50, and PRKAA1 expression, which promotes carcinogenesis. PRKAA1 knockdown in gastric cancer cells showed a significant decrease in cell invasion and migration. It also inhibited the expression of MMP-2 and activation of NF-*κ*B, whereas on the contrary, PRKAA1 involved in NF-*κ*Bp50 mediated gastric cancer cell invasion and migration indicated their role in gastric cell carcinogenesis [56]. *H. pylori*-induced inflammatory response of gastric cells leads to increased epithelial cell turnover by increasing its proliferation and apoptosis. Apart from *H. pylori*, other inflammatory markers such as TNF and interferon-gamma (INF-*γ*) also trigger apoptosis. Another marker identified as *H. pylori* activated peripheral blood mononuclear cells (PBMCs) upregulates the expression of Fas antigen in RGM-1 (Rat Gastric Mucosal Cell First) gastric cells. In the presence of Fas ligand, RGM-1 cells and PBMC medium showed immense and rapid cell proliferation and cell death (Table 1) [29, 57, 58].

Increased gastric mucosal cell turnover also increases the demand for a DNA repair system. Increased cell proliferation results in increased rates of mutation, hence requiring greater surveillance and rectification by DNA mismatch repair (MMR) [59]. Therefore, decreased MMR activity results in mutation. Microsatellite instability (MSI) is a marker of deficiency of DNA MMR activity (Figure 1). Mutations in hMSH3 and hMSH6 (DNA MMR gene), receptors of growth factors, and transforming growth factor *β*-RII are seen in MSI-positive gastric carcinoma [60–62]. Other DNA MMR gene, hMLH1, and sometimes hMSH2 expression are completely lost [63–67]. These findings are enough to suggest that *H. pylori* causes deficient MMR in the gastric mucosal cells, resulting in the development of early stages of gastric carcinoma (Table 1).

### 1.4. Colorectal Cancer and Bacterial Infection

For more than 100 years ago, bacteria were first identified in human tumors. However, the classification of the tumor microbiome remained difficult due to its low abundance [68]. Each type of tumor has a distinct composition of microbiome [68]. The symbiotic relationships between resident microorganisms and the digestive tract contribute significantly to the maintenance of gut homeostasis [69]. Changes to the microbiota triggered by changes in the environment (e.g., infection, diet, and/or lifestyle) may, however, disrupt this symbiotic relationship and facilitate diseases such as inflammatory bowel diseases and cancer. Colorectal cancer is a complex mixture of tumor cells, nonneoplastic cells, and a significant number of microorganisms, and microbiota involvement in colorectal carcinogenesis is becoming increasingly apparent. Nevertheless, several changes in gut microbiota's bacterial composition have been documented in colorectal cancer, indicating a major role for dysbiosis in colorectal carcinogenesis [69]. Some bacterial species, such as *Streptococcus bovis*, *Helicobacter pylori*, *Bacteroides fragilis*, *Enterococcus faecalis*, *Clostridium septicum*, *Fusobacterium spp.*, and *Escherichia coli*, have been identified and suspected to play a role in colorectal carcinogenesis (Table 1) [69]. The potential interactions between bacterial microbiota and colorectal carcinogenesis such as genotoxicity and inflammation derived from bacteria have been found [69].

A microbial etiology for colorectal human cancer (hCRC) has been suggested and pursued for a long time [70]. Establishing how one or more members of the microbiota initiate and/or promote hCRC could stimulate the development of novel prevention approaches, since hCRC has a long time to go from initiation to presentation. It has also been proposed that various intestinal microbes may lead to a common pathway to tumorigenesis [70]. Over 90 percent of hCRC is sporadic, with a small proportion of inherited mutations. Germline mutations in the tumor suppressor gene of adenomatous polyposis coli (APC) are responsible for the family adenomatous polyposis (FAP) (Figure 1) [71]. In addition, at least 80 percent of intermittent hCRC shows adenomatous polyposis coli (APC) mutations as well.

#### 1.4.1. Molecular Mechanism Underlying Induction of Colorectal Cancer

Enterotoxigenic Bacteroides fragilis (ETBF) is a commensal bacterium of the human intestine and a potent initiator of colitis through the secretion of Bacteroides fragilis toxin (BFT) [72] (Table 1). BFT induces the cleavage of E-cadherin in colon cells, which then leads to the activation of NF-*μ*B. Zerumbone, a key component of the plant Zingiber zerumbet (L.), has antibacterial and anti-inflammatory effects. Treatment with zerumbone significantly reduced expression of IL-17A, TNF-*α*, and KC in ETBF-infected mouse colonic tissues [72] (Table 1). Zerumbone-treated ETBF-infected mice also showed a decline in colon NF-*κ*B signalling. Moreover, HT29/C1 colonic epithelial cells treated with BFT-induced BFT signalling and IL-8 secretion. However, an E-cadherin cleavage mediated by BFT was unaffected [72]. It has been found that ETBF colonization in mice did not change after treatment with zerumbone, whereas it decreased ETBF-induced colitis through inhibition of NF-*κ*B signalling [72]. ApcMin mice colonized with the enterotoxigenic human pathobiont *Bacteroides fragilis* (ETBF) as a model of colon tumorigenesis induced by microbes have been used [73]. *Bacteroides fragilis* toxin (BFT) activates a procarcinogenic, multistage inflammatory cascade in colonic epithelial cells (CECs) that includes IL-17R, NF-*κ*B, and Stat3 signals (Figure 1) [73]. While necessary, activation of Stat3 in CECs is not sufficient to cause tumorigenesis of the ETBF colon. Therefore, BFT induces a procarcinogenic signalling relay from the CEC to a Th17 mucosal response resulting in selective NF-*κ*B activation in distal colon CECs, which collectively activates distal colon tumorigenesis based on myeloid cells [73].

While *β*-catenin signalling is documented to be associated with inflammatory responses, and BFT is known to cleave E-cadherin associated with *β*-catenin, little is known about inflammation control in ETBF infection by *β*-catenin mediation [74]. After stimulation with BFT, expression of *β*-catenin in intestinal epithelial cells was reduced relatively early and then recovered relatively late after stimulation to normal levels. In comparison, phosphorylation of *β*-catenin occurred early in stimulation at high rates in BFT-exposed cells and decreased as time went by [74] (Table 1). Inactivation of *β*-catenin in BFT-stimulated cells has resulted in increased NF-*κ*B activity and interleukin-8 (IL-8) expression (Figure 1). In addition, glycogen synthase kinase 3*β* inhibition was associated with increased *β*-catenin expression and attenuated NF-*κ*B activity and expression of IL-8 in BFT-exposed cells. These findings indicate negative control of *β*-catenin in BFT-stimulated intestinal epithelial cells as a consequence of acute inflammation in ETBF infection [74].

Colonic mucosa has been observed in patients with family adenomatous polyposis (FAP), who develop benign precursor lesions (polyps) early in life [75]. Patchy bacterial biofilms predominantly composed of Escherichia coli and Bacteroides fragilis were identified. Genes for colibactin (clbB) and Bacteroides fragilis toxin (BFT), which encode secreted oncotoxins, have been highly enriched in the colonic mucosa of patients with FAP compared to healthy people [75]. It has been found that a tumor colonized with E. coli (colibactin) and enterotoxigenic B. fragilis has demonstrated increased colonial interleukin-17 and colonic epithelial DNA damage with faster tumor initiation and increased mortality compared to mice with a bacterial strain alone (Table 1 and Figure 1). This study indicated an unlikely link between the early colon neoplasia and tumorigenic bacteria [75].

### 1.5. Breast Cancer and Bacterial Infection

The most common cancer among women is breast cancer [76]. Breast cancer is a major cause of death among women all over the world. Breast cancer has a lifetime effect on one in eight women. The number of newly diagnosed cases of invasive breast cancer in the US is estimated at 268,000 in 2019, while the newly diagnosed cases in situ are estimated at about 62,930 [77]. Of these, 41,760 women are expected to die of breast cancer in the US in 2019 [77]. In developed countries, breast cancer survival for five years is over 80 percent thanks to screening services and the consequent early detection [78].

#### 1.5.1. Molecular Mechanism Underlying Induction of Breast Cancer

Breast cancer is characterized by dysbiosis, an aberrant composition of the microbiome [79]. In this study, we address differences in the metabolism of breast cancer cells, as well as breast and gut microbiome composition in breast cancer. The role of the breast microbiome in breast cancer is unclear, but the gut microbiome does seem to play a part in the disease pathology. The gut microbiota secretes bioactive metabolites that modulate breast cancer (reactivated estrogens, short-chain fatty acids, amino acid metabolites, or secondary bile acids) (Figure 1) [79]. Such blood-borne microbial metabolites have been shown to modulate breast cancer behavior. These metabolites mimic human hormones, since they are formed in a “gland” (in this case, the microbiome) and are then transferred through the bloodstream to distant sites of action. These metabolites tend to be essential tumor microenvironmental constituents [79].

While there are proven risk factors for diet, age, and genetic predisposition, most breast cancers have unknown etiologies. The human microbiota is a group of microbes that inhabit the human body. Microbial imbalance, or microbial dysbiosis, has been involved in numerous human diseases including obesity, diabetes, and colon cancer [80]. In a qualitative breast microbiota DNA study, the bacterium *Methylobacterium radiotolerans* has been found to be relatively enriched in tumor tissue, while the *Sphingomonas yanoikuyae* bacterium is relatively enriched in paired normal tissue (Table 1). In paired normal breast tissue, but not in tumor tissue, the relative abundances of these two bacterial species were inversely correlated, indicating that dysbiosis is associated with breast cancer. In addition, total bacterial DNA load was decreased in the tumor versus paired normal and healthy breast tissues. Interestingly, the bacterial DNA load was associated inversely with advanced disease, a result that may have broad implications in breast cancer diagnosis and staging. Microbial DNA is present in the breast and can affect the local immune system [80].

### 1.6. Bladder Cancer and Bacterial Infection

Numerous laboratory, clinical, and community-based epidemiological studies have been conducted to determine the connection between urinary tract bacterial infection and bladder carcinoma incidence. Increased risk of bladder cancer following bacterial urinary tract infection has been identified in patients with recurrent or chronic cystitis and paraplegic patients [81]. Bacteria that are present in the urine have the ability to reduce ingested nitrates into nitrite which transforms into a nitrosating agent in acidic or neutral pH. About 39 to 66% of patients hospitalized with bladder carcinoma tested positive for bacteriuria, thus indicating urinary tract infection (UTI) [82]. In another study, urine samples were collected from 76 bladder carcinoma patients, and bacterial counts were 10^3^ CFU/ml in 60% of patients which was much higher than female patients. Microbial urine profile revealed the presence of *Staphylococcus albus* hemolytic, *Staphylococcus aureus*, *Klebsiella* spp., *Proteus mirabilis*, and *E. coli* [83].

#### 1.6.1. Mechanism of Induction of Bladder Cancer

These species are bacteria-producing nitrate and thus play an important role in the production of N-nitrosamines (Figure 1). These organisms have been shown *in vitro* to perform a nitrosation reaction between ingested or metabolically derived nitrates and secondary amines under physiological pH, leading to the formation of N-nitrosamines (Figure 1) [84]. The formation of endogenous N-nitrosamines leads to the initiation of neoplastic events in patients. Moreover, elevated levels of N-nitrosamines have been consistently detected in bladder carcinoma patients [85]. Bacteria-infected rats have shown also increased nitrosation of amine precursors leading to increased levels of N-nitrosamines [85]. The presence of these compounds in urine may therefore provide the origin of initiating events crucial to the development of bladder cancer (Table 1). However, in order to communicate their carcinogenic effects, these compounds need activation to produce the reactive chemical species that can alkylate constituents of tissue. DNA methylation has been identified exclusively in patients with bladder cancer in different tissues of the human population [86, 87].

### 1.7. Conclusion

It is concluded that various specific species of bacteria have the pathogenic ability to induce carcinogenesis. Although there are some common mechanisms like the release of free radicals that cause damage to DNA and other regulatory proteins, there are other complex molecular mechanisms showing the role of bacterial proteins in the activation of specific inflammatory proteins. Therefore, in this review, we have highlighted the role of bacteria in the induction of malignancy providing evidences of their mechanism. Strong evidence from the literature showed an association of *Salmonella typhi*, *Chlamydia pneumonia*, and *H. pylori* with gallbladder cancer, lung cancer, and gastric cancer, respectively. Therefore, it is increasingly apparent that dissection of the complex interplay between man and microbial flora is essential to understand the pathogenesis of many malignancies.

## Figures and Tables

**Table 1:** Types of cancer induction and mechanisms of carcinogenesis induced by different microbiota.

Cancer	Bacteria inducing cancer	Mechanisms of carcinogenesis	References
Gallbladder cancer	*Salmonella typhi*	Changes in the sequence of p53 gene; activation of protein kinase; cytolethal distending toxin B (CdtB); biliary deoxycholate; cholic acid derivatives; 5-alpha,6-alpha-epoxide cholesterol; upregulation of the PI3K pathway	[8, 10–13]
Lung cancer	*Chlamydia pneumoniae*	Alteration in apoptosis and/or cell programming signalling; overexpression of miRNA-328; by stimulating lung-resident *γδ* T cells; development of Myd88-dependent IL-1b and IL-23; generation of reactive oxygen species; increased secretion of cytokines, IL-8, IL-10, and TNF	[5, 19–24]
Colorectal cancer	*Streptococcus bovis*, *Helicobacter pylori*, *Bacteroides fragilis*, *Enterococcus faecalis*, *Clostridium septicum*, *Fusobacterium spp.*, and *Escherichia coli*	Secretion of Bacteroides fragilis toxin; activation of NF-*μ*B; expression of IL-17A, and TNF-*α*; *β*-catenin expression, induction of IL-17R, NF-*κ*B, and Stat3 signals; induction of the gene expression of colibactin (clbB) and Bacteroides fragilis toxin (BFT), increased colonial interleukin-17, and colonic epithelial DNA damage	[69, 72–75]
Breast cancer	*Methylobacterium radiotolerans*, *Sphingomonas yanoikuyae*	Microbiota secretes bioactive metabolites including estrogens, short-chain fatty acids, amino acid metabolites, or secondary bile acids; dysbiosis	[78, 79]
Bladder cancer	*Staphylococcus albus hemolytic*, *Staphylococcus aureus*, *Klebsiella spp.*, *Proteus mirabilis*, and *E. coli*	Formation of N-nitrosamines; DNA methylation; reactive chemical species	[83–87]
